# Clinical and Microbiological Characteristics of Culture-Positive, Influenza-Associated Pulmonary Aspergillosis: A Single-Center Study in Southern Taiwan, 2016–2019

**DOI:** 10.3390/jof8010049

**Published:** 2022-01-04

**Authors:** Chi-Jung Wu, Cong-Tat Cia, Hsuan-Chen Wang, Chang-Wen Chen, Wei-Chieh Lin, Jen-Chieh Lee, Po-Sheng Chen, Chih-Cheng Hsieh, Wei-Ting Li, Po-Lan Su, Xin-Min Liao, Ming-I Hsieh, Pui-Ching Choi, Wen-Chien Ko

**Affiliations:** 1 National Institute of Infectious Diseases and Vaccinology, National Health Research Institutes, Tainan 70403, Taiwan; wucj@nhri.edu.tw (C.-J.W.); 930505@nhri.edu.tw (H.-C.W.); heaven_ava@nhri.edu.tw (M.-I.H.); pcchoi@nhri.edu.tw (P.-C.C.); 2 Department of Internal Medicine, Division of Infectious Diseases, National Cheng Kung University Hospital, College of Medicine, National Cheng Kung University, Tainan 70456, Taiwan; 3 Department of Internal Medicine, Division of Critical Care Medicine, National Cheng Kung University Hospital, College of Medicine, National Cheng Kung University, Tainan 70456, Taiwan; ctcia@mail.hosp.ncku.edu.tw (C.-T.C.); cwchen@mail.ncku.edu.tw (C.-W.C.); wclin@mail.ncku.edu.tw (W.-C.L.); jclee.eric@msa.hinet.net (J.-C.L.); mdcps027@gmail.com (P.-S.C.); cchsieh520@gmail.com (C.-C.H.); d035289@mail.hosp.ncku.edu.tw (W.-T.L.); 4 Department of Medicine, College of Medicine, National Cheng Kung University, Tainan 70456, Taiwan; 5 Department of Internal Medicine, Division of Chest Medicine and Respiratory Care, National Cheng Kung University Hospital, College of Medicine, National Cheng Kung University, Tainan 70456, Taiwan; polan.750317@gmail.com (P.-L.S.); atmosphere0411@gmail.com (X.-M.L.)

**Keywords:** influenza, aspergillosis, *Aspergillus flavus*, *Aspergillus fumigatus*, *Aspergillus terreus*, azole resistance, bronchoscopy, galactomannan, radiology, tracheobronchitis, Taiwan

## Abstract

This study delineated the characteristics of 24 (11.2%) culture-positive, influenza-associated pulmonary aspergillosis (IAPA) patients out of 215 patients with severe influenza during 2016–2019 in a medical center in southern Taiwan. Twenty (83.3%) patients did not have EORTC/MSG-defined host factors. The mean time from influenza diagnosis to *Aspergillus* growth was 4.4 days, and 20 (83.3%) developed IAPA within seven days after influenza diagnosis. All patients were treated in intensive care units and all but one (95.8%) received mechanical ventilation. *Aspergillus* tracheobronchitis was evident in 6 (31.6%) of 19 patients undergoing bronchoscopy. Positive galactomannan testing of either serum or bronchoalveolar lavage was noted in all patients. On computed tomography imaging, IAPA was characterized by peribronchial infiltrates, multiple nodules, and cavities superimposed on ground-glass opacities. Pure *Aspergillus* growth without bacterial co-isolation in culture was found in 17 (70.8%) patients. *A. fumigatus* (15, 62.5%), *A. flavus* (6, 25.0%), and *A. terreus* (4, 16.7%) were the major causative species. Three patients had mixed *Aspergillus* infections due to two species, and two had mixed azole-susceptible and azole-resistant *A. fumigatus* infection. All patients received voriconazole with an all-cause mortality of 41.6%. Of 14 survivors, the mean duration of antifungal use was 40.5 days. In conclusion, IAPA is an early and rapidly deteriorating complication following influenza that necessitates clinical vigilance and prompt diagnostic workup.

## 1. Introduction

While being sporadically reported decades ago, influenza-associated pulmonary aspergillosis (IAPA) has been recognized as one of the major complications following influenza to date [1]. It was reported in 16–23% of patients with severe influenza in Belgium, the Netherlands, and Taiwan [1,2,3,4]. Notably, only 27–32% of IAPA patients had classical host factors for invasive pulmonary aspergillosis (IPA) defined by the European Organization for Research and Treatment of Cancer and the Mycoses Study Group (EORTC/MSG), and 25–30% were previously healthy [1,2]. After careful assessment in a Dutch–Belgian multicenter study in 2018, influenza has been identified as an independent risk factor for IPA, and the incorporation of influenza as one of the host factors in defining IPA is thus considered appropriate [1]. Nevertheless, according to the EORTC/MSG definition, the classification of probable IPA requires the presence of at least one host factor, clinical feature, and piece of mycological evidence, whereas influenza is not included as a host factor [5]. Moreover, based on the *AspICU* algorithm, which was proposed for diagnosing IPA in critically ill patients without EORTC/MSG host factors, the classification of putative IPA requires a positive culture for *Aspergillus* in bronchoalveolar lavage fluid (BALF) without simultaneous bacterial growth, and therefore, patients suspected to have IPA but with bacterial co-isolation or not receiving bronchoscopic studies would remain unclassified [6]. Facing an unmet diagnostic need and to facilitate clinical studies, Paul E. Verweij et al. recently proposed a case definition of IAPA (referred to as the Amsterdam IAPA criteria herein) in 2020, which includes two disease categories, i.e., invasive *Aspergillus* tracheobronchitis (ATB) and IAPA without ATB, based on expert consensus [7].

Clinical features of IAPA have been described in many studies [1,4,8]. In general, it usually presented as an early and critical complication requiring intensive care following influenza infection with a high mortality rate (49–61%) [1,2,8]. However, the microbiological characteristics of *Aspergillus* from IAPA were addressed to a lesser extent, and the optimal treatment duration for IAPA remains to be determined as the current recommendation of a 6–12 weeks’ treatment duration for IPA is mainly based on the data from immunocompromised patients [9].

In Taiwan, a single-center study reported a high incidence (16.9%) and mortality rate (66.7%) of IPA among severe influenza patients during 2015–2016, and subsequently called physicians’ attention towards this life-threatening complication herein [4]. With the issue of a new case definition and increased vigilance towards IAPA, we aimed to describe the clinical and laboratory characteristics and treatment outcome of IAPA and identify clinical and laboratory clues for the detection of IAPA and its optimal treatment duration. 

## 2. Materials and Methods

This study enrolled adults aged ≥20 years with influenza infection and temporally related isolation of *Aspergillus* species from respiratory samples, including sputum, endotracheal aspirate (ETA), or BALF at National Cheng Kung University Hospital (NCKUH), a tertiary medical center in southern Taiwan, during 2016–2019. Clinical, laboratory, bronchoscopic, and radiological data of eligible patients obtained from medical chart review were analyzed. The diagnosis and classification of IAPA were made according to the Amsterdam IAPA criteria as well as those of EORTC/MSG and *AspICU* [5,6,7]. Early onset IAPA was considered when *Aspergillus* isolates were recovered within seven days after detection of influenza.

In this study, influenza virus infection was impressed based on a positive result of the reverse transcriptase polymerase chain reaction (RT-PCR) test according to the World Health Organization protocol or a rapid antigen test for influenza A and B (BD Veritor™ System) for nasopharyngeal or throat swab, sputum, or BALF [10]. For severe influenza patients with pulmonary infiltrates, fungal cultures of sputum and/or ETA were performed and the serum galactomannan (GM) index was determined by the Platelia *Aspergillus* Ag assay (Bio-Rad, Marnes-la-Coquette, France). Bronchoscopic examination, which allows visualization of the large airways and obtaining BALF for fungal culture and GM testing, was recommended for critically ill influenza patients with suspected IPA due to either clinical deterioration, suspicious radiographic lesions, mold isolated from sputum and/or ETA, or positive serum GM testing, and the attending physicians in charge of patient care and pulmonologists who performed bronchoscopy made the final decision. According to the Amsterdam’s IAPA criteria, the sample was considered positive at a cut-off index >0.5 for serum and ≥1.0 for BALF. Consecutive *Aspergillus* isolates from each patient were collected and stored at −80 °C until use. They were identified based on morphology, sequence analysis of the internal transcribed spacer region and calmodulin gene along with additional phylogenetic analysis for cryptic species, and subjected to antifungal susceptibility testing for minimum inhibitory concentration (MIC) determination following the CLSI M38-A2 method [11,12]. For azole-resistant *Aspergillus fumigatus* (ARAF) isolates, the *cyp51A* gene was analyzed [13]. The inter- and intra-patient genetic relatedness of *A. fumigatus*, *Aspergillus flavus*, and *Aspergillus terreus* isolates were determined by microsatellite genotyping as previously described [14,15,16].

Using the SPSS statistics (version 17.0. Chicago: SPSS Inc.), univariate and multivariate analyses were performed to identify the independent factors associated with all-cause in-hospital mortality among IAPA patients. In univariate analyses, categorical variables were compared by the Fisher’s exact test or chi-squared test, and continuous variables by *t*-test; in multivariate analysis, binary logistic regression model was used. A *p* value of less than 0.05 was considered statistically significant, and all tests were two-tailed.

## 3. Results

During 2016–2019, 31 patients with laboratory-confirmed influenza infection and isolation of *Aspergillus* species from respiratory sample(s) with a temporal relationship were identified. Based on the Amsterdam IAPA criteria, 24 patients were classified as proven (case 1) or probable (*n* = 23, case 2–24) cases of IAPA, with 3, 6, 6, and 9 patients occurring in 2016, 2017, 2018, and 2019, respectively. Seven patients who did not meet the IAPA definition were not included for further analyses. During the study period, a total of 215 cases of severe influenza requiring intensive care were identified, and thus the prevalence of culture-positive IAPA was 11.2% (24/215) in those with severe influenza. Clinical and laboratory characteristics of 24 IAPA patients are presented in Table 1, Table 2, and Table S1. Sixteen (66.7%) of 24 cases of IAPA had more than one respiratory sample with *Aspergillus* growth, and thus a total of 52 *Aspergillus* isolates were recovered from 24 patients. Their antifungal susceptibility profile is provided in Table 3.

Of 24 IAPA patients, four (16.7%) had EORTC/MSG host factors, and five (20.8%) were classified as proven or probable IPA based on the EORTC/MSG definition, while ten (41.7%) were classified as proven or putative IPA based on the *AspICU* definition. Eighteen (75%) patients were infected by influenza A (subtype H1: 11, 45.8% and H3: 7, 29.2%) and six (25%) by influenza B, confirmed by PCR in all but one case, in whom a positive rapid antigen test was noted. Of 19 patients in whom *Aspergillus* were recovered after influenza diagnosis, the mean time from influenza diagnosis to *Aspergillus* growth was 4.4 days (interquartile range (IQR) 1–6 days), whereas in the remaining five patients, influenza testing was performed after the notification of *Aspergillus* growth. All patients were treated in intensive care units (ICUs), and all but one (23, 95.8%) needed mechanical ventilation, among whom 15 (65.2%) had their first *Aspergillus*-positive culture either shortly prior to endotracheal intubation (3 patients: 1–2 days earlier) or immediately within one day after intubation (12 patients).

**Figure 1:**
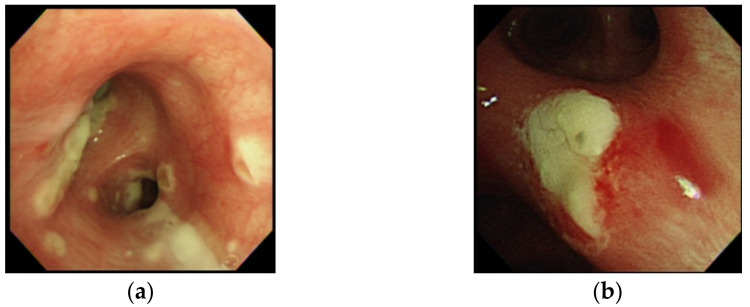
Bronchoscopic examination in case 17 with influenza-associated pulmonary aspergillosis (IAPA) due to *A. fumigatus*, which demonstrated vesicles and whitish patches over bilateral bronchial tress (**a**), and in case 23 with IAPA due to *A. flavus*, which demonstrated an area of whitish plague over left secondary carina that was difficult to remove (**b**), but resolved under voriconazole in a follow-up bronchoscopic examination eight days later.

Twenty (83.3%) patients had a serum GM index >0.5. Of 19 patients undergoing bronchoscopy, 15 (78.9%) patients had a serum GM index >0.5 and 14 (73.7%) had a BALF GM index ≥1.0, which included all four patients with a serum GM index <0.5. Therefore, all 24 patients with IAPA had either a serum GM index >0.5 or a BALF GM index ≥1.0. Via bronchoscopy, ATB presenting as tracheobronchial ulceration, pseudomembrane, or whitish plaque was noted in six patients (31.6%) (Figure 1), and in whom their BALF GM index was ≥1.0. The BALF GM index in the six patients with ATB was higher than that in the thirteen patients without ATB, though the difference was not significant (4.3 vs. 3.0, *p* = 0.17). 

Of 19 patients with both ETA and BALF available for fungal cultures, nine (47.4%) had *Aspergillus* growth in BALF. All 19 patients had *Aspergillus* growth in ETA, which was obtained at a mean time of 2.4 (IQR 1–4) days earlier than the collection of BALF. Prior antifungal use did not decrease the fungal culture yield of BALF, as two of ten patients with BALF obtained before and seven of nine with BALF obtained after antifungal therapy had *Aspergillus* growth (20.0% vs. 77.8%, *p =* 0.04). Most (21, 87.5%) patients had a single *Aspergillus* species recovered from respiratory samples (13 *A. fumigatus*, 5 *A. flavus*, and 3 *A. terreus*), while three patients had concurrent isolation of two *Aspergillus* species (*A. fumigatus* plus *A. pseudonomius*, *A. fumigatus* plus *A. flavus*, and *A. terreus* plus *A. allahabadii*) (Table 4). Overall, *A. fumigatus* (15, 62.5%) was the most common cause of IAPA, followed by *A. flavus* (6, 25.0%), and *A. terreus* (4, 16.7%). Microsatellite genotypes of the isolates of three major species differed among patients, indicating no clonal spread (Table 4). Of fifteen patients with multiple isolates of the same species recovered, six (46.7%) harbored multiple microsatellite genotypes of one *Aspergillus* species, including *A. fumigatus* isolates belonging to 2–4 genotypes in four patients and *A. flavus* isolates belonging to two genotypes in two patients. Notably, a pure growth of *Aspergillus* without bacterial growth in the culture plate was found in seventeen (70.8%) patients, while co-isolation of pathogenic bacteria was found in five (20.8%) patients and co-isolation of commensal flora in two (8.3%) patients. Ten (41.7%) patients had bacterial pulmonary or bloodstream co-infections 0–7 days prior to the recovery of *Aspergillus*, and *Klebsiella pneumoniae* in four cases was the most common bacterial co-pathogen (Table S1).

**Figure 2:**
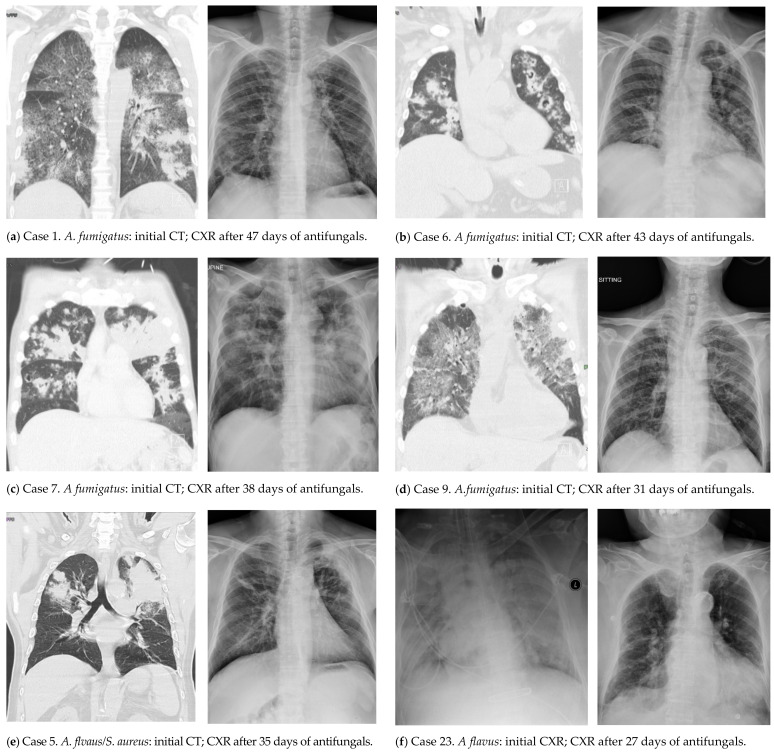
Initial (**left**) and follow-up (**right**) radiological images of six patients with influenza-associated aspergillosis who survived.

Thirteen patients underwent chest computed tomography (CT) scanning (Figure 2), in which peribronchial infiltrate (13, 100%), multiple nodules (10, 76.9%), wedge-shaped consolidation (8, 61.5%), and cavities (4, 30.8%) were noted. Ground-glass opacity (GGO) was present in seven (53.8%) patients, among whom five of six patients tested for *Pneumocystis jirovecii* PCR had a negative result in a respiratory sample, suggesting a low possibility of *Pneumocystis* colonization or infection. Of eleven patients with CXR only, patchiness and/or consolidation were noted in all patients and nodules in three patients.

All patients received anti-influenza agents (oseltamivir or preramivir) and voriconazole. The mean interval from the growth of *Aspergillus* (the day when the respiratory samples were collected for fungal cultures) to the initiation of antifungal therapy was short (4.0 days) and did not significantly differ between survivors and non-survivors (3.4 days vs. 4.9 days, *p =* 0.52). Azole-resistant *A. fumigatus* isolates were identified in two cases (no. 17 and 21). Case 17 started voriconazole treatment two days before the growth of both azole-susceptible and azole-resistant *A. fumigatus* isolates (voriconazole MIC: 2 μg/mL, isavuconazole MIC: 16 μg/mL; wild-type *cyp51A*) from ETA, but eventually died of IAPA after 18 days’ voriconazole and subsequent 12 days’ liposomal-amphotericin B treatment. Case 21 started voriconazole treatment two days after the growth of azole-susceptible *A. fumigatus* from ETA, but an azole-resistant *A. fumigatus* isolate (voriconazole MIC: 4 μg/mL; TR34/L98H mutation in *cyp51A*) was detected from ETA five days later. The patient eventually recovered with voriconazole therapy for 25 days without relapse. 

The all-cause in-hospital and IAPA-attributable mortality rate was 41.6% (10/24) and 37.5% (9/24), respectively, among 24 IAPA patients. Univariate and multivariate analyses were not able to identify factors independently related to a fatal outcome. Among 14 survivors (13 without EORTC/MSG host factors), antifungal treatment was continued until there was no more growth of *Aspergillus* spp. in respiratory samples and a resolution or stabilization of pulmonary lesions in chest radiographs (Figure 2). The mean duration of antifungal use in 14 survivors was 40.5 (IQR 32–51) days and nine (64.3%) received voriconazole for ≤six weeks. Of 14 survivors, one patient died of lymphoma three months later, and ten patients receiving ≥six-month follow-up (nine receiving ≥nine-month follow-up) in the study hospital remained free from aspergillosis, among whom the mean duration of antifungal use was 42.1 (IQR 34–51) days and six received antifungal for ≤six weeks.

## 4. Discussion

This study delineated the clinical and laboratory characteristics of 24 patients with culture-positive IAPA at a medical center based on the Amsterdam IAPA criteria, which was composed of a combination of bronchoscopic, mycological, and radiological findings. Overall, we found that IAPA might occur in the absence of classical host factors, be caused by a mixture of *Aspergillus* isolates, and manifest as an early onset, rapidly deteriorating but treatable complication following influenza infection.

By analyzing culture-positive cases, the prevalence of IAPA in severe influenza patients was noted to be at least 11.2% here, which was within the range of those reported in the Dutch–Belgian study (19.2%), Switzerland (11.1%), Spain (7.2%), Canada (7.2%), China (5.4%), and a recent report from Taiwan (19.9%) [1,17,18,19,20,21]. As reported earlier, IAPA occurred not only following influenza A (both subtypes H1 and H3), but also influenza B [1]. The short interval from the influenza diagnosis to *Aspergillus* growth (4.4 days) was similar to that observed in Canada (5 days) and the U.S. (6 days) [19,22]. Notably, the first *Aspergillus*-positive cultures in about two-thirds (65.2%) of patients were noted shortly before or immediately after intubation, suggestive of the acquisition of *Aspergillus* pathogens before intubation. These findings also indicated that IAPA developed shortly following influenza when both mucosal and systemic immune defenses against inhaled airborne *Aspergillus* conidia were compromised by the influenza virus [7,22]. The vast majority of IAPA patients required ICU care and mechanical ventilation, as noted in earlier studies (89–100%) [1,3,8,17]. Taken together, IAPA was an early and rapidly deteriorating complication following influenza. 

As delayed antifungal treatment is associated with an unfavorable outcome, early diagnosis and early antifungal therapy are crucial for IAPA management [2]. Clinical presentations of IAPA are non-specific, and thus the diagnosis of IAPA is usually dependent on a series of diagnostic tests, including, in order of invasiveness, serum GM testing, fungal cultures of sputum and/or ETA, chest CT, and bronchoscopy, which are all incorporated in the Amsterdam IAPA criteria [7]. This proposed definition seems more clinically feasible, as only 20.8% and 41.7% of IAPA patients herein were classified as IPA based on the EORTC/MSG and *Asp*ICU definitions, respectively. The merits of the Amsterdam IAPA criteria include that the classical EORTC/MSG host factors are not required, and those without bronchoscopic study or with bacterial co-isolation from BALF could be properly defined.

Previous studies reported a higher sensitivity of GM testing in BALF (88–94%) than in serum (65–78%) for detecting IAPA [1,2,22]. Though the sensitivity of GM testing in BALF (73.7%) was found to be slightly lower than in serum (83.3%) here, BALF GM testing remained important and was complementary to serum GM testing, as the former identified all four IAPA patients with negative serum GM. Moreover, our study revealed four microbiological features that were rarely addressed before. First, for IAPA, ETA samples had a higher culture yield than BALF samples. Together with a high sensitivity of serum GM testing, routine serum GM testing and fungal culture from ETA for patients with severe influenza upon ICU admission might aid the early detection of IAPA. A positive test result should promptly trigger diagnostic workup for invasive aspergillosis. Second, about 70% of IAPA patients showed pure growth of *Aspergillus* on culture plates without bacterial co-isolation, indicating that a dominant growth of *Aspergillus* in respiratory samples is associated with invasive fungal disease. The pure growth of *Aspergillus* also echoes the mycological criterion for putative IPA in the *Asp*ICU algorithm, i.e., semiquantitative *Aspergillus* growth in BALF without bacterial growth. Nevertheless, bacterial co-isolation was not uncommon in influenza patients, as seen in 29.2% of IAPA patients, and the drawback of the *Asp*ICU algorithm in such a scenario could be overcome by the Amsterdam IAPA criteria. Third, *A. fumigatus* was almost exclusively reported as the cause of IAPA in western countries [1,2,8,19,22], but *A. flavus* or *A. terreus* could be etiologic agents of IAPA here. *A. flavus* has been recognized to cause human aspergillosis particularly in Asia, the Middle East, and Africa, and was reported as the cause in 3 out of 10 culture-positive IAPA patients in China [23,24]. Though less common, IPA due to *A. terreus* occurred in certain geographical regions, such as Austria [25]. This study also identified less-common *Aspergillus* species, including *A. pseudonomius* (belonging to *Aspergillus* section *Flavi)* and *A.* *allahabadii* (*Aspergillus* section *Terrei*). Albeit rare, *A. pseudonomius* has been reported to cause human disease [26], while human infections due to *A.* *allahabadii* have not been identified yet. The pathogenic role of these uncommon species remained to be elucidated, since they co-existed with other well-recognized pathogenic *Aspergillus* species (*A. fumigatus* or *A. terreus*) here. Finally, mixed *Aspergillus* infections due to different species, microsatellite genotypes, or azole susceptibility profiles were not uncommon in IAPA, suggesting exposure to diverse *Aspergillus* conidia in the environment. Such a finding echoed the international guideline recommending antifungal susceptibility testing of multiple colonies (up to five) from a single culture [27]. Moreover, the discovery of ARAF due to the *Aspergillus* isolate with an environmental resistance mechanism (TR_34_/L98H mutation) following the initial azole-susceptible isolate in case 21 underlined the necessity of repeated susceptibility testing in the consecutive *Aspergillus* isolates, because the subsequent acquisition of environmental resistant isolates remains possible.

Our study and a French study shared a similar prevalence rate of ATB (31.6% and 28.6%, respectively) among IAPA patients, and both revealed that immunocompromised as well as immunocompetent patients were at risk for ATB [8]. We additionally found that *A. flavus* and *A. terreus* could be the causes of ATB, in addition to *A. fumigatus* reported in France. However, the association between ATB and a higher BALF GM level or mortality revealed in France was not found here, probably because of our limited number of cases.

The common CT images in patients with IAPA due to *A. fumigatus* herein included peribronchial infiltrates, multiple nodular consolidation, and cavitary lesions superimposed on areas of GGO. The appearance of GGO might be explained by the underlying viral pneumonia, as was seen in 45% of patients with H1N1 infection on CT imaging [28]. The features of peribronchial infiltrates reflect the pathogenic process of IAPA, where *Aspergillus* spreads along the tracheobronchial trees and eventually leads to ATB and multiple nodules and cavities within the lung parenchyma. Multiple nodules and cavities have been recognized as radiological characteristics of IAPA, and occurred less frequently in influenza patients without IPA [24]. These CT features in combination are regarded as radiological clues for IAPA.

The all-cause mortality rate of 24 IAPA patients with voriconazole therapy was 41.6%. Of note, case 21 survived with voriconazole treatment despite a high voriconazole MIC (4 μg/mL) of the causative *A. fumigatus* isolate. This favorable outcome might be explained by high voriconazole concentrations in pulmonary epithelial lining fluid (EFL), based on an average ELF-to-plasma ratio of 11 [29]. A relapse of IPA was not observed among ten survivors with available follow-up information who received a mean of six-week antifungal treatment and among six who received antifungal for ≤six weeks, and the treatment duration was shorter than that of 6–12 weeks recommended for IPA in patients with EORTC/MSG host factors. Furthermore, the resolution or stabilization of pulmonary lesions on chest radiographs could also be achieved with ≤six weeks antifungal treatment here. Therefore, our data suggested that antifungal therapy for six weeks might be sufficient for some IAPA patients without classical immunocompromised conditions.

Our study has several limitations. First, only culture-positive patients were enrolled, and thus true IAPA patients were considered for analysis. Culture-negative patients with positive GM results in blood or BAL were not included. With this restrictive criterion adopted herein, the incidence of IAPA is likely to be underestimated. Second, steroid use was previously found to be independently associated with IPA [3], but our study design cannot allow us to elucidate the role of steroid use in IAPA development. However, about two-thirds of IAPA patients did not have prior steroid exposure, indicating that steroid use might not be the only predisposing factor contributing to IAPA. Third, CT imaging was not performed in patients with monomicrobial IAPA due to *A. flavus* and *A. terreus*, so it is not clear whether both species presented similar CT findings as *A. fumigatus*. Finally, the case number of IAPA was limited. Further studies enrolling more cases are warranted to elucidate unanswered questions, such as the role of less-common *Aspergillus* species in IAPA and the prognostic impact of lymphopenia and steroid use during IAPA treatment, and to confirm the appropriateness of six-week antifungal therapy for IAPA patients without EORTC/MSG host factors. 

## 5. Conclusions

Our study revealed that IAPA is an early and rapidly deteriorating complication of influenza that might occur in the absence of underlying disease and be caused by a mixture of *Aspergillus* isolates. Routine serum GM testing and fungal culture from ETA for patients with severe influenza upon ICU admission could be considered for early recognition of IAPA. A positive test result should promptly trigger diagnostic workup for invasive aspergillosis. Growth of pathogenic *Aspergillus* species without bacterial co-isolation, tracheobronchitis in a bronchoscopic study, and typical CT findings are useful clues for diagnosing IAPA. A high level of clinical vigilance, prompt diagnosis, and early treatment can ensure a better outcome of this dangerous but treatable complication following influenza.

## Figures and Tables

**Table 1:** Characteristics of 24 patients with influenza-associated pulmonary aspergillosis (IAPA).

Clinical Variables	Case No. (%) or Mean ± Standard Deviation			Univariate, *p* Value
	All, *n* = 24	Survivors, *n* = 14	Non-Survivors, *n* = 10	
Baseline characteristics				
Age (years)	67.6 ± 14.5	69.2 ± 14.2	65.3 ± 15.2	0.526
Male sex	13 (54.2)	7 (50)	6 (60)	0.697
Body mass index > 25 kg/m^2^	11 (45.8)	5 (35.7)	6 (60)	0.408
Active smoking	5 (20.8)	3 (21.4)	2 (20)	>0.999
Chronic heart failure	4 (16.7)	2 (14.3)	2 (20)	>0.999
Chronic kidney disease ^a^	6 (25.0)	3 (21.4)	3 (30)	0.665
Diabetes mellitus	11 (45.8)	6 (42.9)	5 (50)	>0.999
Known risk factors for invasive aspergillosis				
Chronic obstructive pulmonary disease	4 (16.7)	3 (21.4)	1 (10)	0.615
Liver cirrhosis	1 (4.2)	1 (7.1)	0 (0)	>0.999
EORTC/MSG host factor	4 (16.7)	1 (7.1)	3 (30)	0.272
Hematological malignancy	3 (12.5)	1 (7.1)	2 (20)	0.550
Active solid tumor	2 (8.3)	1 (7.1)	1 (10)	>0.999
Steroids use before *Aspergillus* growth				
≥10 mg/day for ≥3 days in the past 14 days	8 (33.3)	4 (28.6)	4 (40)	0.673
≥0.3 mg/kg/day for ≥3 weeks in the past 60 days ^b^	3 (12.5)	1 (7.1)	2 (20)	0.550
Influenza				
Influenza A	18 (75)	10 (71.4)	8 (80)	>0.999
A (H1)	11 (45.8)	6 (42.9)	5 (50)	-
A (H3)	7 (29.2)	4 (28.6)	3 (30)	-
Influenza B	6 (25.0)	4 (28.6)	2 (20)	>0.999
*Aspergillus*				
*Aspergillus* only in respiratory samples	17 (70.8)	9 (64.3)	8 (80.0)	0.653
*A. fumigatus*	15 (62.5)	9 (64.3)	6 (60)	>0.999
*A. flavus*	6 (25.0)	4 (28.6)	2 (20)	>0.999
*A. terreus*	4 (16.7)	2 (14.3)	2 (20)	>0.999
*A. pseudonomius*	1 (4.2)	0 (0)	1 (10)	0.833
*A. allahabadii*	1 (4.2)	1 (7.1)	0 (0)	>0.999
Diagnostics				
Tracheobronchitis by bronchoscopy	6/19 (31.6)	2/10 (20.0)	4/9 (44.4)	0.517
Serum galactomannan index >0.5	20 (83.3)	11 (78.6)	9 (90)	0.615
BAL galactomannan index ≥ 1.0	14/19 (73.7)	8/10 (80)	6/9 (66.7)	0.889
Proven/probable by EORTC/MSG definition	5 (20.8)	2 (14.3)	3 (30)	0.615
Proven/probable by *AspICU* definition	10 (41.7)	5 (35.7)	5 (50)	0.678
Clinical and laboratory data				
Early onset IAPA	20 (83.3)	12 (85.7)	8 (80.0)	>0.999
APACHE II score ^d^	16.8 ± 9.6	13.8 ± 8.6	21.0 ± 9.7	0.064
Neutropenia (neutrophil < 500/μL)	0 (0)	0 (0)	0 (0)	n/a
Lymphopenia (lymphocyte < 1000/μL)	22 (91.7)	13 (92.9)	9 (90%)	>0.999
Lymphocyte count (/μL)	518.8 ± 278.2	538.0 ± 252.8	491.9 ± 322.7	0.698
Bacterial co-infection	10 (41.7)	5 (35.7)	5 (50)	0.678
Days from influenza diagnosis to *Aspergillus* growth, *n* =19 (11, 8) ^e^	4.4 ± 4.0	3.3 ± 3.6	5.9 ± 4.2	0.163
Steroids use 0–10 days after *Aspergillus*	12 (50)	6 (42.9)	6 (60)	0.680
Steroids doses (mg) 0–10 days after *Aspergillus, n* = 12 (6, 6)	303.8 ± 130.9	271.3 ± 179.8	336.3 ± 53.5	0.416
Hemodialysis	7 (29.2%)	2 (14.3%)	5 (50%)	0.085
Mechanical ventilation	23 (95.8)	13 (92.9)	10 (100)	>0.999
Mechanical ventilation days after *Aspergillus*, *n* = 23 (13,10)	16.5 ± 11.0	14.0 ± 10.5	20.1 ± 11.3	*n*/a
Antimicrobial therapy				
Anti-influenza agent before *Aspergillus*	14 (58.3)	7 (50)	7 (70)	0.421
Use of anti-*Aspergillus* antifungal agent	24 (100)	14 (100)	10 (100)	n/a
Days from *Aspergillus* growth to initiation of antifungals	4.0 ± 5.7	3.4 ± 2.3	4.9 ± 8.5	0.523
Duration of antifungal treatment (days)	31.7 ± 15.9	40.5 ± 12.8	19.3 ± 10.7	n/a
All-cause in-hospital mortality	10 (41.7)	n/a	n/a	n/a
IAPA-attributable mortality	9 (37.5)	n/a	n/a	n/a

Abbreviation: APACHE, Acute Physiology and Chronic Health Evaluation; BAL: bronchoalveolar lavage; n/a, non-applicable. ^a^ Glomerular filtration rate < 60 mL/min/1.73 m^2^. ^b^ EORTC/MSG criteria. Three patients had mixed infection due to two *Aspergillus* species (please see the text for details). ^d^ APACHE II score was calculated on the day when *Aspergillus* spp. was first isolated. ^e^ Only the data of 19 cases in whom influenza tests were performed prior to the isolation of *Aspergillus* were counted.

**Table 2:** Clinical and laboratory data of 24 patients with proven or probable influenza-associated pulmonary aspergillosis based on the Amsterdam IAPA criteria.

Case	Age (y)/Sex	Underlying Conditions	Flu Type	Days from Influenza Diagnosis to *Aspergillus* Growth	Serum GM	BAL GM/ATB	*Aspergillus* spp.	Bacterial Co-Isolation	CT (CXR) Findings	Sequential Antifungals (Days)	Outcome (Days after *Aspergillus*)
1	40+/M	CS, pancreatitis	B	6	2.13	nd	*A. fumigatus*	no	GGO, PBI, N, ws-C	VRC (5), CPF (5), VRC (36)	alive
2	60+/F	CAD, DM, ESRD on HD	A (H1)	1	7.89	0.18/n	*A. fumigatus*	NF	GGO, PBI, C	VRC (63)	alive
3	70+/M	PPU	B	5	0.19	2.99/y	*A. fumigatus*	no	P	VRC (12), LAmB (15)	died (30)
4	40+/M	no	A (H1)	0	2.83	6.72/n	*A. terreus*	no	(N, C)	VRC (34)	alive
5	70+/M	CAD, CHF, DM	A (H1)	0	0.36	3.16/n	*A. flavus*	SA	PBI, N, ws-C	VRC (51)	alive
6	70+/M	CKD, COPD, MGUS	B	4	4.26	5.07/n	*A. fumigatus*	no	PBI, N, cavities	VRC (51)	alive
7	50+/M	DM	A (H3)	6	0.71	5.25/n	*A. fumigatus*	no	GGO, PBI, N, cavities, ws-C	VRC (42)	alive
8	70+/F	CAD, DM, HTN	A (H3)	5	0.55	0.32/n	*A. fumigatus*	no	PBI, C	VRC (17)	died (44)
9	70+/M	CHF, CS-E, HBV/LC, HCC, lymphoma	A (H3)	1	0.09	2.44/n	*A. fumigatus*	no	GGO, PBI, N, ws-C	VRC (28)	alive
10	80+/M	Old TB	B	5	1.09	1.07/y	*A. fumigatus,* *A. pseudonomius*	no	GGO, PBI, N, cavities, ws-C	VRC (27)	died (34)
11	70+/F	DM, HTN	B	0	1.63	nd	*A. fumigatus*	KP	(P)	VRC (32)	alive
12	80+/F	COPD, CS, old TB, HCV	B	5	3.55	nd	*A. fumigatus*	no	(P)	VRC (18)	alive
13	70+/F	CKD	A (H3)	2	6.26	6.18/n	*A. fumigatus*, *A. flavus*	no	GGO, PBI, N, ws-C	VRC (59)	alive
14	40+/M	ALL s/p HSCT, HBV	A (H3)	13	4.13	5.17/y	*A. fumigatus*	no	PBI, N, ws-C	VRC (2), VRC+CPF (6)	died (7)
15	60+/F	Sarcoma s/p doxorubicin, CS-E	A (H1)	1	5.57	6.53/n	*A. terreus*	no	(P)	VRC (21)	died (21)
16	80+/F	COPD, HTN	A (H1)	10	0.56	4.98/y	*A. terreus*, *A. allahabadii*	PA	(P)	VRC (42)	alive
17	70+/M	COPD, CHF, CS, DM, HTN	A (H1)	11	4.40	5.04/y	azole-S, -R *A. fumigatus*	no	PBI, N, cavities	VRC (18), LAmB (12)	died (31)
18	90+/F	DM, HTN	A (H3)	1	4.57	nd	*A. flavus*	NF	(P)	VRC (35)	alive
19	30+/M	DM, ESRD on HD	A (H1)	6	1.01	0.09/n	*A. terreus*	no	(P)	VRC (7)	died (8)
20	70+/F	CHF, CKD, CS, DM, HTN, RA	A (H3)	4	2.87	0.51/n	*A. flavus*	PA	(N, P)	VRC (5), LAmB (4), VRC (13), AmB (14)	died (37), unrelated
21	60+/M	CS, DM, HTN	A (H1)	8	0.56	0.19/n	azole-S, -R *A. fumigatus*	no	GGO, PBI, N, C	VRC (25)	alive
22	50+/M	HBV	A (H1)	0	5.78	nd	*A. fumigatus*	no	(P, C)	VRC (3)	died (4)
23	80+/F	HTN	A (H1)	4	0.35	6.65/y	*A. flavus*	no	(P, N, ws-C)	VRC (42)	alive
24	80+/F	DM, MM, CS-E	A (H1)	3	3.63	2.16/n	*A. flavus*	AB	(N, C)	VRC (18)	died (22)

Abbreviation: AB, *Acinetobacter baumannii;* ALL, acute lymphoblastic leukemia; AmB, amphotericin B deoxycholate; ATB, *Aspergillus* tracheobronchitis; C, consolidation; CAD, coronary artery disease; CHF, congestive heart failure; CKD, chronic kidney disease; COPD, chronic obstructive pulmonary disease; CPF, caspofungin; CS, corticosteroids; CS-E, corticosteroids fulfilling EORTC/MSG criteria; DM, diabetes mellitus; ESRD, end-stage renal disease; F, female; GGO, ground glass opacity; HBV/LC, chronic hepatitis B/liver cirrhosis; HCC, hepatocellular carcinoma; HCV, chronic hepatitis C; HD, hemodialysis; HSCT, hematological stem cell transplantation; HTN, hypertension; KP, *Klebsiella pneumoniae*; LAmB, liposomal amphotericin B; M, male; MM, multiple myeloma; MGUS, monoclonal gammopathy of unknown significance; N, nodules; nd, not done; NF, normal flora; P, patchiness; PA, *Pseudomonas aeruginosa*; PBI, peribronchial infiltrates; PPU, perforated peptic ulcer; R: resistant; RA, rheumatoid arthritis; S: susceptible; SA, *Staphylococcus aureus*; TB, tuberculosis; VRC, voriconazole; ws-C, wedge-shaped consolidation.

**Table 3:** Antifungal susceptibility profiles of 52 *Aspergillus* isolates determined by CLSI M38-A2.

*Aspergillus* spp. (No. of Isolate)	MIC or MIC Range (Geometric Mean) (μg/mL)				
	Amphotericin B	Itraconazole	Voriconazole	Posaconazole	Isavuconazole
*Aspergillus* section *Fumigati*					
*A. fumigatus* (33)	0.25–1 (0.62)	0.12->16 (0.57)	0.25–4 (0.64)	0.03–1 (0.12)	nd
azole-susceptible (31)	0.25–1 (0.63)	0.12–0.5 (0.44)	0.25–1 (0.59)	0.03–0.25 (0.11)	nd
azole-resistant (2)					
*cyp51A* (wild-type) (1) ^a^	0.5	>16	2	1	16
*cyp51A* (TR_34_/L98H) (1) ^b^	0.5	>16	4	1	8
*Aspergillus* section *Flavi*					
*A. flavus* (11)	1–2 (1.12)	0.12–0.5 (0.20)	0.5–1 (0.50)	0.06–0.25 (0.12)	nd
*A. pseudonomius* (2)	1 (1)	0.5 (0.5)	1 (1)	0.25 (0.25)	1 (1)
*Aspergillus* section *Terrei*					
*A. terreus* (5)	1 (1)	0.12 (0.12)	0.43 (0.25–0.5)	0.08 (0.03–0.12)	nd
*A. allahabadii* (1)	2	0.5	2	0.12	2

Abbreviations: CLSI, Clinical and Laboratory Standards Institute; MIC, minimum inhibitory concentration; nd, not done; ^a^ from case 17; ^b^ from case 21.

**Table 4:** *Aspergillus* isolates recovered from 24 patients with influenza-associated pulmonary aspergillosis and microsatellite genotypes of *A. fumigatus*, *A. flavus*, and *A. terreus* isolates.

Case	Year of Isolation	Day of Culture	Sample	Species	Microsatellite Genotype									No. of Genotype/No. of Isolate
					2A	2B	2C	3A	3B	3C	4A	4B	4C	
IAPA due to *A. fumigatus*														
1	2016	D0	sputum	*A. fumigatus*	23	20	25	30	13	50	5	11	5	1/2
		D0	lung	*A. fumigatus*	23	20	25	30	13	50	5	11	5	
2	2016	D0	ETA	*A. fumigatus*	24	9	8	0	12	7	10	11	10	1/1
3	2016	D0	ETA	*A. fumigatus*	22	20	18	23	11	26	12	10	10	2/2
		D5	BAL	*A. fumigatus*	18	22	13	43	12	14	14	9	10	
6	2017	D0	sputum	*A. fumigatus*	22	12	17	34	20	16	10	9	5	4/4
		D1	sputum	*A. fumigatus*	15	21	17	8	19	16	11	8	5	
		D2	ETA	*A. fumigatus*	25	20	14	11	7	18	12	7	5	
		D6	BAL	*A. fumigatus*	15	21	17	35	19	16	12	8	0	
7	2017	D0	sputum	*A. fumigatus*	20	27	18	29	12	30	22	11	5	1/3
		D1	ETA	*A. fumigatus*	20	27	18	29	12	30	22	11	5	
		D4	ETA	*A. fumigatus*	20	27	18	29	12	30	22	11	5	
8	2017	D0	ETA	*A. fumigatus*	26	22	11	31	11	23	13	11	8	1/3
		D7	ETA	*A. fumigatus*	26	22	11	31	11	23	13	11	8	
		D16	ETA	*A. fumigatus*	26	22	11	31	11	23	13	11	8	
9	2017	D0	ETA	*A. fumigatus*	23	19	20	19	11	17	17	17	10	1/2
		D1	BAL	*A. fumigatus*	23	19	20	19	11	17	17	17	10	
10	2018	D4	BAL	*A. fumigatus*	13	19	11	35	24	8	10	9	8	1/1
		D4	ETA	*A. pseudonomius*										
		D4	ETA	*A. pseudonomius*										
11	2018	D0	ETA	*A. fumigatus*	20	19	13	38	24	14	14	10	8	1/1
12	2018	D0	ETA	*A. fumigatus*	26	19	15	32	15	7	10	11	8	1/1
14	2018	D0	ETA	*A. fumigatus*	25	19	19	26	13	16	10	18	10	1/3
		D1	BAL	*A. fumigatus*	25	19	19	26	13	16	10	18	10	
		D3	ETA	*A. fumigatus*	25	19	19	26	13	16	10	18	10	
17	2019	D0	ETA	*A. fumigatus* ^a^	24	16	13	28	16	23	6	12	10	4/4
		D0	ETA	*A. fumigatus*	23	16	8	30	33	18	8	9	13	
		D7	ETA	*A. fumigatus*	20	22	15	28	14	23	10	9	5	
		D7	ETA	*A. fumigatus*	20	19	13	38	24	14	14	10	8	
21	2019	D0	ETA	*A. fumigatus*	15	21	8	18	27	9	17	11	13	2/2
		D5	ETA	*A. fumigatus* ^b^	18	24	14	32	19	31	16	9	5	
22	2019	D0	ETA	*A. fumigatus*	20	18	28	44	12	7	9	11	5	1/2
		D1	ETA	*A. fumigatus*	20	18	28	44	12	7	9	11	5	
IAPA due to *A. flavus*														
5	2017	D0	ETA	*A. flavus*	21	11	8	9	14	4	7	9	9	1/1
18	2019	D0	ETA	*A. flavus*	17	11	21	17	12	13	7	7	11	2/2
		D2	ETA	*A. flavus*	26	11	12	12	26	9	9	5	9	
20	2019	D0	ETA	*A. flavus*	16	11	12	8	31	14	8	7	10	1/2
		D2	ETA	*A. flavus*	16	11	12	8	31	14	8	7	10	
23	2019	D0	BAL	*A. flavus*	18	7	15	8	21	9	6	9	9	2/2
		D1	ETA	*A. flavus*	16	14	10	8	20	14	8	9	10	
24	2019	D0	ETA	*A. flavus*	44	16	10	8	13	14	5	7	37	1/1
IAPA due to *A. fumigatus* and *A. flavus*														
13	2018	D2	ETA	*A. flavus*	29	11	11	8	7	10	7	12	10	1/3
		D2	ETA	*A. flavus*	29	11	11	8	7	10	7	12	10	
		D3	BAL	*A. flavus*	29	11	11	8	7	10	7	12	10	
				*A. fumigatus*	16	15	10	8	48	15	5	9	7	1/2
				*A. fumigatus*	16	15	10	8	48	15	5	9	7	
IAPA due to *A. terreus*														
4	2017	D4	BAL	*A. terreus*	14	9	27	5	7	66	8	24	7	1/1
15	2018	D0	ETA	*A. terreus*	8	11	26	11	8	7	10	9	8	1/2
		D3	BAL	*A. terreus*	8	11	26	11	8	7	10	9	8	
16	2019	D0	ETA	*A. terreus*	13	12	24	9	8	14	8	11	7	1/1
		D6	ETA	*A.allahabadii*										
19	2019	D0	ETA	*A. terreus*	12	9	23	4	7	11	8	11	5	1/1

Abbreviation: BAL, bronchoalveolar lavage; “D0” and “Dn” indicate the day of and *n* days after the first isolation of *Aspergillus* species; ETA, endotracheal aspirate. ^a^ Azole-resistant *A. fumigatus* with wild-type *cyp51A*. ^b^ Azole-resistant *A. fumigatus* with TR_34_/L98H mutation in *cyp51A*.

## Data Availability

The data that support the findings of this study are available on request from the corresponding author.

## Supplementary Materials

The following supporting information can be downloaded at: https://www.mdpi.com/article/10.3390/jof8010049/s1, Table S1: Supplementary clinical and laboratory data of 24 patients with influenza-associated pulmonary aspergillosis.
