# Supplementary material for: Clinical and Microbiological Characteristics of Culture-Positive, Influenza-Associated Pulmonary Aspergillosis: A Single-Center Study in Southern Taiwan, 2016–2019
Source: J Fungi (Basel). 2022 Jan 4;8(1):49. doi: 10.3390/jof8010049 (PMC8780730; doi:10.3390/jof8010049)
Supplement: Supplementary file 1 [file jof-08-00049-s001.zip › jof-1533147-supplementary.pdf]

Journal of *Fungi*

MDPI

Table S1. Supplementary clinical and laboratory data of 24 patients with influenza-associated pulmonary aspergillosis.

Case	Steroid use 0-60 days before *Aspergillus*, mg/kg/d (days)	Steroid use 0-10 days after *Aspergillus*, mg/kg/d (days)	Days from intubation to *Aspergillus* growth	Co-infection 0-7 day before *Aspergillus* growth	Days from *Aspergillus* growth to antifungals	RRT	MV (days)	Outcome
1	1.45 (6)	0.51 (4)	no intubation	no	3	n	0	alive
2	n	n	<1	no	5	y	14	alive
3	n	n	5	no	4	n	35	died
4	n	n	<1	no	3	n	21	alive
5	n	n	<1	SA BSI/PN	2	n	7	alive
6	n	n	−2	no	5	n	14	alive
7	n	n	−1	KP BSI/PN	> 5	n	35	alive
8	n	n	5	KP PN	> 5	n	23	died
9	0.44 (33)	0.67 (10)	<1	PJP	4	n	5	alive
10	n	0.65 (10)	4	KP BSI	> 5	n	38	died
11	n	n	<1	KP PN	> 5	n	7	alive
12	0.97 (5)	0.37 (10)	5	SA PN	2	n	9	alive
13	n	n	−2	no	2	y	5	alive
14	n	0.61 (8)	<1	no	0	y	7	died
15	1.21 (25)	n	<1	PM BSI	1	n	21	died
16	n	0.44 (10)	<1	PA PN	2	n	27	alive
17	0.64 (13)	0.48 (10)	<1	no	−2	n	24	died
18	n	1.0 (10)	<1	no	2	n	5	alive
19		n	5	no	2	y	13	died
20	0.16 (60)	0.48 (10)	<1	PA PN	2	y	11	died, unrelated
21	0.15 (3)	0.18 (10)	8	no	2	n	20	alive
22	n	0.59 (5)	<1	no	2	y	4	died
23	n	n	4	no	0	n	27	alive
24	0.52 (59)	1.17 (5)	3	AB PN	4	y	25	died

Abbreviations: AB, *Acinetobacter baumannii*; BSI, bloodstream infection; KP, *Klebsiella pneumoniae*; MV, mechanical ventilation; PA, *Pseudomonas aeruginosa*; PM, *Proteus mirabilis*; PN, pneumonia; PJP, *Pneumocystis jerovecii* pneumonia; RRT, renal replacement therapy; SA, *Staphylococcus aureus*.
